# The Prevalence of Anemia and Moderate-Severe Anemia in the US Population (NHANES 2003-2012)

**DOI:** 10.1371/journal.pone.0166635

**Published:** 2016-11-15

**Authors:** Chi Huu Hong Le

**Affiliations:** Department of Biology, Emory University, Atlanta, Georgia, United States of America; University of Florida, UNITED STATES

## Abstract

Since anemia is associated with poor health outcomes, the prevalence of anemia is a significant public health indicator. Even though anemia is primarily caused by iron deficiency, low oxygen-carrying capacity may result from other conditions such as chronic diseases, which remain a relevant health concern in the United States. However, studies examining current rates of anemia in the total US population and in more specific subgroups are limited. Data from five National Health and Nutrition Examination Surveys (NHANES) from 2003 to 2012 were analyzed to assess two outcomes: anemia and moderate-severe anemia, which were based upon serum hemoglobin levels (Hb) as per World Health Organization (WHO) definitions. Statistical analysis using SAS examined temporal trends and the prevalence of anemia among sexes, age groups, and races/ethnicities. The study estimated that an average of 5.6% of the U.S. population met the criteria for anemia and 1.5% for moderate-severe anemia during this 10-year period. High-risk groups such as pregnant women, elderly persons, women of reproductive age, non-Hispanic blacks, and Hispanics were identified, and relationships between multiple risk factors were examined. Rates of anemia in men increased monotonically with age, while that of women increased bimodally with peaks in age group 40–49 years and 80–85 years. The effect of risk factors was observed to compound. For instance, the prevalence of anemia in black women aged 80–85 years was 35.6%, 6.4 times higher than the population average. Moreover, anemia is a growing problem because of the increased prevalence of anemia (4.0% to 7.1%) and moderate-severe anemia (1.0% to 1.9%), which nearly doubled from 2003–2004 to 2011–2012. Thus, these results augment the current knowledge on anemia prevalence, severity, and distribution among subgroups in the US and raised anemia as an issue that requires urgent public health intervention.

## Introduction

Although anemia is a global public health problem, updated data on the prevalence of anemia in the general United States (US) population is not yet available. Due to the reduced oxygen-carrying capacity, anemia has serious health implications that affect both morbidity and mortality [1, 2]. Symptoms of anemia range from fatigue and weakness to reduced cognitive performance [3]. Anemic older adults have increased hospitalization and mortality rates [1]. In congestive-heart failure patients, anemia is a common condition (17%) and results in significantly worse functioning capacity and survival rates [4, 5]. Moreover, anemia has been shown to impair cognitive and psychomotor development in children [6–8]. Iron-deficiency anemia has been found to increase the likelihood of pre-term labor, abnormally low birth weight, and maternal mortality when severe [9–11]. Thus, at-risk groups are children, pregnant women, women of reproductive age, and the elderly [12, 13]. Another potential risk factor is race/ethnicity. Studies have found that anemia is 3 times more common in African Americans than in Whites [14]. While iron deficiency is the leading cause for anemia, the reduced count of red blood cells can arise from other causes such as chronic diseases, which are growing concerns for public health [15]. Therefore, anemia remains a relevant health problem requiring a more comprehensive understanding on its impact in the US. In order to update current knowledge on the epidemiology of anemia, this study examined the prevalence of anemia overall and by severity level in the general US population between 2003 and 2012. This analysis further investigated periodic trends and prevalence within particular subgroups: age, sex, and race/ethnicity.

## Methods

### Study Data and Sample

The National Health and Nutrition Examination Survey (NHANES), which was conducted every 2-year period by the Center for Disease Control, provided cross-sectional health, nutrition, and health behavior data of U.S. non-institutionalized civilian population [16]. In order to provide a nationally representative sample, the generality of data was retained by the survey’s stratified multistage clustered probability sampling strategy, in which narrowing selection by geographical location, households, and individuals was made [16]. More regular surveys have been conducted to characterize population health in the US since 1999. For every survey, around 10,000 participants from 30 selected counties out of approximately 3,000 U.S. counties were asked to participate in a household interview, subsequent physical examinations and laboratory tests at the mobile examination center (MEC) [16]. Certain subgroups (non-Hispanic black, Mexican-American, low-income white, and older persons) were oversampled to increase the reliability of data; however, sample weights measures accounted for readjustment of subgroups’ proportions within the general population [16]. Informed consent was given by participants as per NHANES protocol [16]. Sampling and data collection protocol were approved by National Center for Health Statistics institutional review board [16].

For the primary analysis, data sets from five NHANES cycles between 2003 and 2012 were examined. Only participants with available data pertaining to anemia and potential risk factors (age, sex, race/ethnicity) were included. Subjects with missing results on laboratory tests for defining anemia or no response on age, sex, and race/ethnicity were excluded. The resulting sample consisted of 41,026 individuals between ages 0.5 and 85 years. Males and females were approximately equally represented in the sample. Women with known positive pregnancy status (776) were excluded from the general analysis and were examined separately. Within the analyzed sample, 67.1% were non-Hispanic white, 11.8% non-Hispanic black, 14.7% Hispanic, and 6.4% were “other.” NHANES data were anonymized before access and analysis for this study.

### Study Variables

The definition of anemia was based on serum hemoglobin (Hb) threshold (g/dL) in Table 1 as recommended by the World Health Organization (WHO) [17]. There are three levels of anemia categorized by the WHO: mild, moderate, and severe [17]. Due to the small number of anemic participants with higher severity level, the moderate and severe groups were combined to form a moderate-severe category in Table 1. Moreover, anemia refers to all participants with serum Hb below mild anemia threshold, regardless of severity (Table 1).

**Table 1:** Hemoglobin thresholds (g/dL) to define anemia for population groups.

Population group	Non-anemia	Anemia	Moderate-severe anemia
Children (0.5–4.9 years)	≥11	<11	<10
Children (5.0–11.9 years)	≥11.5	<11.5	<11
Children (12.0–14.9 years)	≥12	<12	<11
Non-pregnant women (≥15 years)	≥12	<12	<11
Pregnant women	≥11	<11	<10
Men (≥15 years)	≥13	<13	<11

From the available demographic data, population groups were further categorized as follows: preschool-age children (0.5–4.9 years), school-age children (5.0–11 and 12–14 years), pregnant women (no age range defined, positive pregnancy status), women of reproductive age (female, 15–49 years), men (male, 15–29, 30–39, 40–49, and 50–59 years), and elderly persons (60–69, 70–79 and 80–85 years). Race/ethnicity subgroups were non-Hispanic white, non-Hispanic black, Hispanic, and others, which are hereafter referred to as white, black, Hispanic, and others.

### Analysis

Data analysis was completed using SAS (Statistical Analysis System, version 9.2; SAS Institute, Cary, NC, USA). Subjects’ characteristics were examined using mean procedure (Proc surveymeans) and frequency procedure (Proc surveyfreq), which had weighted percentage and 95% confidence interval (CI). Prevalence of anemia and moderate-severe anemia were analyzed by age groups, sex, race/ethnicity, and cohort survey year. Differences in demographic statistics were tabulated using the chi-square test. As mentioned previously, women with positive pregnancy status were examined separately. The race/ethnicity category “others” was also excluded from further comparisons by race/ethnicity. Due to the large number of—examined characteristics relative to the sample size, age groups of male participants were not stratified by race for the moderate-severe anemia group.

The analysis also accounted for NHANES complex sampling design by including sampling frame information (primary sampling units and strata) and the mobile examination center/home examination final weights in all analyses. Since the sample combined data from multiple surveys, the ten-year sample weights were calculated in accordance to NHANES analytic guidelines [9]. The sample weights were applied to calculate the proportions of subjects with anemia and moderate-severe anemia. Plots of anemia rates by age, sex, and race/ethnicity were also produced. Distributions of serum hemoglobin level (Hb) by sex were also graphed.

## Results

### Prevalence of anemia

Within the total sample, the overall prevalence of anemia in the US population was 5.6% with 95% confidence interval of 5.1–6.1% (Table 2). In addition, the rate of moderate-severe anemia was 1.5%, and the 95% CI was 1.4–1.7% (Table 2). These measurements excluded pregnant women.

**Table 2:** Characteristics of the study population and prevalence of anemia and moderate-severe anemia by age group, gender, race/ethnicity and cohort survey year, NHANES 2003–2012.

	Study population			Anemia		Moderate severe anemia		
	N	Weighted %*	n	Weighted prevalence (%)^†^	95% CI	n	Weighted prevalence (%)^†^	95% CI
**Total**	41,026	100.0	2,957	5.6	5.1–6.1	745	1.5	1.4–1.7
**Age group (years)**								
0.5–4	3,532	4.1	137	3.4	2.6–4.3	21	0.5	0.2–0.8
5–11	5,581	8.5	165	2.0	1.5–2.5	43	0.5	0.3–0.8
12–14	2,778	4.2	136	3.3	2.5–4.1	24	0.6	0.3–1.0
15–29	8,591	20.6	505	3.8	3.2–4.5	138	1.2	0.9–1.5
30–39	4,012	13.8	262	4.8	4.2–5.5	86	1.6	1.3–2.0
40–49	4,266	15.9	368	6.5	5.5–7.5	150	2.4	1.9–2.9
50–59	3,736	14.1	227	4.4	3.6–5.2	48	1.2	0.8–1.6
60–69	3,971	9.4	372	6.5	5.1–7.8	77	1.3	0.9–1.8
70–79	2,757	5.9	409	12.4	10.6–14.2	81	2.4	1.8–2.9
80–85	1,802	3.4	376	19.4	17.2–21.6	77	4.0	2.8–5.2
**Gender**								
Female	20,307	50.6	1,896	7.6^‡^	6.9–8.3	600	2.5^‡^	2.2–2.8
Male	20,719	49.4	1,061	3.5^‡^	3.0–3.9	145	0.5^‡^	0.4–0.6
**Race/ ethnicity**								
Non-Hispanic White	16,157	67.1	797	4.0	3.5–4.5	172	0.9	0.8–1.1
Non-Hispanic Black	9,826	11.8	1,351	14.9	13.7–16.1	348	4.3	3.9–4.8
Hispanic	12,156	14.7	600	5.1	4.4–5.8	172	1.7	1.4–2.1
Others	2,887	6.4	209	6.1	5.0–7.3	53	1.9	1.1–2.6
**Cohort survey year**								
2003–2004	8,128	19.5	454	4.0^‡^	3.2–4.8	99	1.0^‡^	0.7–1.3
2005–2006	8,046	19.5	461	4.5^‡^	3.8–5.2	112	1.1^‡^	0.8–1.3
2007–2008	8,217	19.9	597	5.8^‡^	4.2–7.4	162	1.8^‡^	1.3–2.3
2009–2010	8,734	20.3	646	6.3^‡^	5.5–7.0	162	1.7^‡^	1.5–2.0
2011–2012	7,901	20.9	799	7.1^‡^	5.6–8.6	210	1.9^‡^	1.5–2.4

Note: *The weighted percentages reflect the proportion of the study population, survey weighted to the US population.

^†^The weighted prevalence percentages reflect the prevalence of total anemia or moderate-severe anemia, survey weighted to the US population. The primary analysis was limited to subjects without pregnancy as defined in the Methods section (N = 41,026).

^‡^p for the column prevalence difference within each variable of interest <0.0001.

### Sex

**Fig 1:**
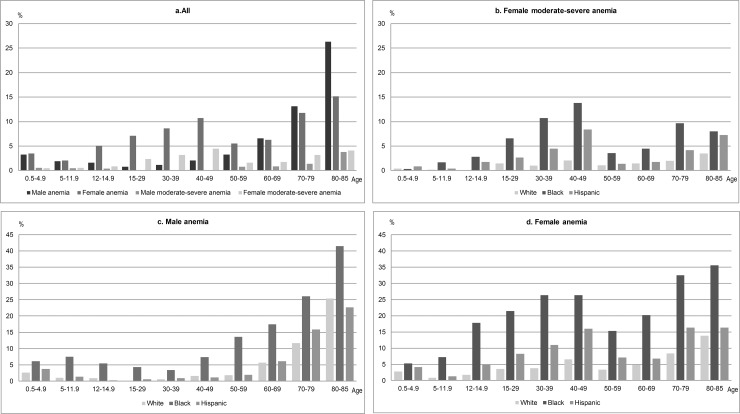
Prevalence of anemia and moderate-severe anemia by gender, age group, and race/ethnicity, NHANES 2003–2012. *The weighted prevalence percentages reflect the prevalence of total anemia and moderate-severe anemia, survey weighted to the US population. The analysis was limited to subjects without pregnancy as defined in the Methods section (N = 41,026): Non-Hispanic White (N = 16,157), Non-Hispanic Black (N = 9,826) and Hispanic (N = 12,156).

On average, the prevalence of anemia in non-pregnant females was significantly higher than that of males. Excluding positive pregnancy status, the proportion of anemic females was twice that of males (7.6% vs. 3.5%, p<0.0001, Table 2). Regarding severity, moderate-severe anemia was 5 times more common in non-pregnant females in comparison to males (2.5% vs. 0.5%, p<0.0001, Table 2). The exception to this pattern is the age group of 80–85 years, in which twice as many males as females had anemia (26.3% vs. 15.2%, p<0.0001, Table 3). However, both sexes for this age group had approximately equivalent rates of moderate-severe anemia (3.8% and 4.1%, p = 0.7398, Table 3). The relationship between sex and age was also noted. While anemia prevalence in males increased monotonically with age from 0.8% for age group 15–29 years to 26.3% for age group 80–85 years, it increased bimodally in females before and after age of 50 years (Table 3, Fig 1). For females, the bimodal distribution also held true for moderate-severe anemia.

**Table 3:** Prevalence of anemia and moderate-severe anemia by gender and age groups, NHANES 2003–2012.

	Anemia			Moderate severe anemia		
	n	Weighted prevalence (%)*	95% CI	n	Weighted prevalence (%)*	95% CI
**Male**	1,061	3.5	3.0–3.9	145	0.5	0.4–0.6
Age group (years)						
0.5–4	73	3.3	2.3–4.3	12	0.6	0.2–0.9
5–11	81	1.9	1.3–2.6	20	0.5	0.1–0.9
12–14	32	1.6	0.9–2.3	5	0.4	0.0–0.8
15–29	71	0.8	0.5–1.1	5	0.1	0.0–0.2
30–39	36	1.2	0.8–1.6	2	0.1	0.0–0.4
40–49	57	2.1	1.3–2.9	5	0.1	0.0–0.3
50–59	91	3.3	2.3–4.3	13	0.8	0.2–1.3
60–69	187	6.6	4.9–8.4	27	0.9	0.3–1.4
70–79	210	13.1	11.1–15.1	23	1.4	0.7–2.1
80–85	223	26.3	22.7–30	33	3.8	2.5–5.1
**Female**	1,896	7.6	6.9–8.3	600	2.5	2.2–2.8
Age group (years)						
0.5–4	64	3.5	2.2–4.9	9	0.5	0.1–1.0
5–11	84	2.1	1.5–2.7	23	0.6	0.3–0.9
12–14	104	5.1	3.7–6.4	19	0.9	0.4–1.4
15–29	434	7.1	5.9–8.3	133	2.4	1.8–3.0
30–39	226	8.6	7.4–9.8	84	3.2	2.5–3.9
40–49	311	10.7	9.1–12.2	145	4.5	3.5–5.5
50–59	136	5.5	4.3–6.7	35	1.6	1.0–2.1
60–69	185	6.3	4.8–7.9	50	1.8	1.1–2.4
70–79	199	11.8	9.1–14.5	58	3.2	2.2–4.2
80–85	153	15.2	13–17.4	44	4.1	2.5–5.6

Note: *The weighted prevalence percentages reflect the prevalence of total anemia and moderate-severe anemia, survey weighted to the US population. The primary analysis was limited to subjects without pregnancy as defined in the Methods section (N = 41,026).

### Age

Among all age groups, school-age children (5–11 years) had the lowest prevalence of anemia (2.0%, Table 2). Moreover, moderate-severe anemia was least common in pre-school aged children (0.5–4 years, 0.5%) and school-age children (5–11 years, 0.5%, Table 2). The age group of 80–85 years had the highest proportion of anemia (19.4%) and moderate-severe anemia (4.0%, Table 2). Both anemia and moderate-severe anemia rates increased bimodally with peaks at 40–49 age groups (6.5% and 2.4%, respectively) and 80–85 age groups (19.4% and 4.0%, respectively, Table 2).

### Race/ethnicity

The prevalence of anemia and moderate-severe anemia varied among races. For all age groups, blacks had the highest prevalence of anemia for both sexes (Table 4). In general, the proportion of Hispanics with anemia and moderate-severe anemia is higher than that of whites. However the prevalence of anemia in males was observed to be lower but not statistically significant in age groups 12–14 (p = 0.1594) and 40–49 (p = 0.4704) for Hispanics vs. whites (Table 4). The differences between blacks in comparison to whites or Hispanics were by several folds (p<0.0001). For women of reproductive age, anemia was more common in blacks than in whites by 4 to 7 times (p<0.0001), and in Hispanics in comparison to whites, by 2 to 3 times (p<0.0001, Table 4). For females, the disparity was the largest for the age group 30–39 years based on the increased prevalence (6.9 times for blacks vs. whites, p<0.0001, 2.9 times for Hispanics vs. whites, p<0.0001, Table 4). The relation between race/ethnicity subgroups also held for female moderate-severe anemic participants, and the age group 30–39 years had the largest increase in rates (10.7 times for blacks vs. whites, p<0.0001, 4.5 times for Hispanics vs. whites, p<0.0001, Table 4).

**Table 4:** Prevalence of anemia and moderate-severe anemia by age group, gender and race, NHANES 2003–2012.

	Non-Hispanic White			Non-Hispanic Black			Hispanic		
	n	Weighted prevalence (%)*	95% CI	n	Weighted prevalence (%)*	95% CI	n	Weighted prevalence (%)*	95% CI
**Male anemia**	391			444			156		
Age group (years)									
0.5–4	13	2.6	0.9–4.2	29	6.1	4.1–8.2	28	3.7	2.3–5.0
5–11	9	1.0	0.1–1.8	56	7.5	5.6–9.4	14	1.4	0.6–2.1
12–14	3	0.9	0.0–2.0	23	5.5	3.2–7.7	2	0.2	0.0–0.6
15–29	1	0.1	0.0–0.2	56	4.3	2.9–5.7	7	0.6	0.0–1.1
30–39	6	0.6	0.2–1.1	13	3.4	1.6–5.2	6	0.9	0.1–1.7
40–49	18	1.6	0.6–2.5	29	7.4	4.4–10.3	6	1.1	0.2–2.0
50–59	16	1.8	0.8–2.7	57	13.6	10.4–16.7	11	1.9	0.8–3.0
60–69	50	5.7	3.6–7.8	92	17.4	13.0–21.8	34	6.1	4.0–8.2
70–79	107	11.7	9.4–14.0	60	26.0	20.2–31.7	33	15.9	8.8–22.9
80–85	168	25.4	21.3–29.4	29	41.4	28.0–54.9	15	22.7	11.5–33.9
**Female anemia**	406			907			444		
Age group (years)									
0.5–4	10	2.8	1.0–4.6	22	5.3	2.9–7.7	27	4.2	2.2–6.3
5–11	6	0.8	0.1–1.6	57	7.2	5.3–9.2	12	1.3	0.5–2.0
12–14	6	1.8	0.3–3.4	72	17.8	13.5–22.1	22	4.9	2.3–7.4
15–29	57	3.6	2.4–4.9	240	21.5	18.5–24.5	105	8.2	5.9–10.6
30–39	35	3.8	2.5–5.0	103	26.4	22.2–30.7	65	11.0	8.4–13.5
40–49	64	6.6	5.0–8.3	137	26.3	22.0–30.6	86	16.0	12.3–19.6
50–59	26	3.4	2.1–4.8	65	15.3	11.9–18.7	29	7.1	3.1–11.1
60–69	42	4.8	2.9–6.6	93	20.2	16.4–24.1	43	6.8	4.2–9.4
70–79	67	8.4	5.6–11.2	80	32.5	26.1–38.9	37	16.3	10.9–21.7
80–85	93	13.8	11.4–16.1	38	35.6	25.6–45.5	18	16.3	6.5–26.1
**Female moderate severe anemia**	122			279			153		
Age group (years)									
0.5–4	3	0.4	0.0–1.0	2	0.3	0.0–0.8	3	0.9	0.0–2.3
5–11	2	0.2	0.0–0.6	14	1.7	0.8–2.7	4	0.4	0.0–0.9
12–14	0	0.0	NA	11	2.8	1.1–4.6	7	1.8	0.4–3.2
15–29	18	1.5	0.7–2.2	73	6.6	4.9–8.4	31	2.7	1.4–4.0
30–39	10	1.0	0.3–1.6	41	10.7	7.8–13.6	24	4.5	2.6–6.3
40–49	22	2.1	1.0–3.2	70	13.8	10.2–17.4	45	8.4	5–11.8
50–59	8	1.1	0.4–1.9	15	3.6	2.2–5.1	8	1.4	0.4–2.4
60–69	15	1.5	0.7–2.3	20	4.5	2.8–6.3	14	1.8	0.8–2.7
70–79	20	2.0	1.1–3.0	24	9.7	6.4–13	9	4.2	1.3–7.1
80–85	24	3.5	1.9–5.0	9	8.0	2.8–13.1	8	7.3	1.2–13.5

Note: *The weighted prevalence percentages reflect the prevalence of total anemia and moderate-severe anemia, survey weighted to the US population. The analysis was limited to subjects without pregnancy as defined in the Methods section: Non-Hispanic White (N = 16,157), Non-Hispanic Black (N = 9,826) and Hispanic (N = 12,156). NA = Not applicable.

For males, the general trend between different racial/ethnic groups was also observed for the prevalence of anemia. However, the largest increases were seen in the age group 15–29 (43 times for blacks vs. whites, p<0.0001, and 6 times for Hispanics vs. whites, p = 0.0218, Table 4). The analysis excluded examination between race/ethnicity and moderate-severe anemia for male subgroup due to small sample size.

### Survey period

From 2003–2004 to 2011–2012, the overall prevalence of anemia and moderate-severe anemia nearly doubled from 4.0% to 7.1% and 1.0% to 1.9%, respectively (p<0.0001, Table 2). The rates were observed to increase every survey cycle for anemia and moderate-severe anemia with the exception of 2009–2010 period for the latter (Table 2). For both categories of anemia, the largest rates change was observed between 2005–2006 and 2007–2008 periods (29% for anemia and 64% for moderate-severe anemia, Table 2).

During this 10-year period, the highest increase in prevalence was observed in male Hispanics for moderate-severe anemia (200%, Table 5). The lowest rates change was seen in the black females subgroup for moderate-severe anemia (17%, Table 5). Moreover, rates of anemia increased from 5.1% to 11.8% and from 17.5% to 25.0% in black males and females, respectively (Table 5). In all three race/ethnicity groups, males were also seen to have the highest change in prevalence of both categories of anemia during the study period (Table 5).

**Table 5:** Prevalence of anemia and moderate-severe anemia by survey years, gender and race, NHANES 2003–2012.

	Non-Hispanic White			Non-Hispanic Black			Hispanic		
	n	Weighted prevalence (%)*	95% CI	n	Weighted prevalence (%)*	95% CI	n	Weighted prevalence (%)*	95% CI
**Male anemia**	391			444			156		
Survey years									
2003–2004	64	2.1	1.6–2.5	63	5.1	3.5–6.8	25	0.9	0.4–1.4
2005–2006	67	2.6	1.8–3.4	82	7.6	4.9–10.3	14	0.9	0.3–1.5
2007–2008	89	3.2	1.1–5.3	91	9.2	6.6–11.8	33	1.8	1.2–2.5
2009–2010	100	3.7	2.6–4.8	75	8.3	5.8–10.7	46	2.5	1.8–3.2
2011–2012	71	3.6	2.6–4.5	133	11.8	9.2–14.4	38	2.7	1.1–4.3
**Female anemia**	406			907			444		
Survey years									
2003–2004	67	3.7	2.8–4.7	166	17.5	14.1–20.9	53	4.6	1.6–7.5
2005–2006	62	3.9	2.6–5.2	165	17.8	16.1–19.5	52	5.3	3.0–7.5
2007–2008	94	5.6	4.1–7.2	162	20.4	16.2–24.6	110	9.0	6.1–11.9
2009–2010	111	6.1	4.9–7.4	156	21.5	18.9–24.2	124	9.3	7.6–11.0
2011–2012	72	5.0	3.8–6.3	258	25.0	20.8–29.3	105	12.8	10.4–15.3
**Male moderate severe anemia**	50			69			19		
Survey years									
2003–2004	5	0.2	0.0–0.3	9	0.7	0.2–1.1	2	0.1	0.0–0.4
2005–2006	3	0.1	0.0–0.2	13	1.5	0.8–2.2	1	-	0.0–0.1
2007–2008	13	0.5	0.1–1.0	10	1.0	0.4–1.7	5	0.3	0.0–0.6
2009–2010	17	0.7	0.5–1.0	13	1.3	0.8–1.9	5	0.2	0.1–0.4
2011–2012	12	0.6	0.2–1.1	24	1.7	0.9–2.5	6	0.4	0.0–0.9
**Female moderate severe anemia**	122			279			153		
Survey years									
2003–2004	18	1.0	0.4–1.7	47	6.6	4.5–8.7	13	1.4	0.4–2.4
2005–2006	14	0.9	0.3–1.6	54	6.6	5.1–8.1	23	2.8	0.6–5.1
2007–2008	34	2.0	1.3–2.6	50	7.1	5.6–8.6	44	3.7	2.9–4.6
2009–2010	36	2.0	1.4–2.5	49	7.2	6.1–8.3	36	2.7	1.8–3.6
2011–2012	20	1.4	0.5–2.2	79	7.7	5.2–10.1	37	5.0	3.3–6.8

*The weighted prevalence percentages reflect the prevalence of total anemia and moderate-severe anemia, survey weighted to the US population. The analysis was limited to subjects without pregnancy as defined in the Methods section: Non-Hispanic White (N = 16,157), Non-Hispanic Black (N = 9,826) and Hispanic (N = 12,156).

### Pregnant women

Among 776 pregnant women, the prevalence of anemia was 8.8% (95% CI: 5.8–11.9%) and moderate-severe anemia was 3.5% (95% CI: 1.5–5.6%). However, black pregnant women had the highest proportion of anemia (24.2%, 95% CI: 15.1–33.3%) and of moderate-severe anemia (13.6%, 95% CI: 5.3–21.8%). Rates of anemia in whites, Hispanic, and other races were 3.1%, 9.2% and 15.6%, respectively (result not shown).

### Serum hemoglobin (Hb)

**Fig 2:**
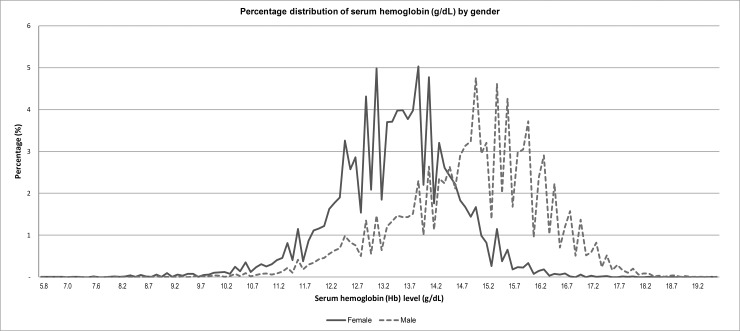
Percentage distribution of serum hemoglobin (g/dL) by gender, NHANES 2003–2012. * Female mean Hb: 13.4 g/dL, 95% CI: 13.4–13.5 g/dL; Male mean Hb: 14.9 g/dL, 95% CI: 14.9–15.0 g/dL. ^†^The weighted percentages reflect the distribution of Hb, survey weighted to the US population. The graph is independent of age, race and ethnicity. The analysis was limited to subjects without pregnancy as defined in the Methods section (N = 41,026), Males (N = 20,719) and Females (N = 20,307).

In the general sample, the distribution of serum hemoglobin (Hb) levels had mean 14.2 g/dL (95% CI: 14.1–14.2) and median 14.1 g/dL (95% CI: 14.0–14.1) (result not shown). Mean Hb levels in females (13.4 g/dL, 95% CI: 13.4–13.5 g/dL) was lower than that in males (14.9 g/dL, 95% CI: 14.9–15.0 g/dL, Fig 2). The distribution for females without positive pregnancy status was observed to be left of the males’ distribution (Fig 2).

## Discussion

Through the analysis of NHANES data from 2003 to 2012, it was estimated that 5.6% (95% CI: 5.1–6.1%) of the US population had anemia and 1.5% (95% CI: 1.4–1.7%) had moderate-severe anemia (Table 2). The WHO categorized public health significance of anemia based on prevalence as follow: normal (≤4.9%), mild (5.0–19.9%), moderate (20.0–39.9%), and severe (≥40%) [17]. Based on the data, anemia in the general U.S. population would be classified as a mild public health problem. However, the study showed that anemia and more severe levels of anemia are serious health concerns for specific subgroups such as blacks, Hispanics, older adults over 60 years old, non-pregnant women of reproductive age, and pregnant women. The identification of these at-risk groups was consistent with previous findings for races [14, 18, 19], elderly persons [13, 20], non-pregnant women of reproductive age and pregnant women [21]. The measured prevalence of anemia in pregnant women (8.8%) was higher but within the 95% confidence interval of the WHO reported rates for the US pregnant women (5.7%, 95% CI: 3.6–8.9%) [22]. The disparities of anemia rates among the sexes were observed through significant difference in prevalence, as well as shifted serum hemoglobin distribution curve. Moreover, these risk factors were observed to compound in effect. For example, the anemia proportion of black women with age 80 to 85 years was 35.6%, which was 6.4 times higher than the population average (Table 4). Black women of reproductive age had an anemia prevalence of 21.5–26.4%, which differed from the population average by 3.8–4.7 times and was also consistent with a 1999–2002 previously measured rates (24.4%) [13]. It was also found that 24.2% of black pregnant women had anemia and 13.6% had moderate-severe anemia, and 9.2% of Hispanic pregnant women met the criteria for anemia. Evidently, anemia is a national health concern that critically needs to be addressed by public health interventions.

The overall prevalence of anemia and moderate-severe anemia monotonically increased and nearly doubled from the years 2003–2004 to 2011–2012. A similar trend for anemia has been reported by several other authors [23, 24]. The proportion of anemia was observed to decrease during the periods of 1988–1994 and 1999–2002 in US women and children and between 1970s and 1985 in children and pregnant women [25, 26]. However, this analysis showed an increasing trend in rates of anemia for both men and women from 2003 to 2012. For example, from 2003 to 2012, the proportion of anemia increased in black males from 5.1% to 11.8% and black females from 17.5% to 25.0%. An increase was also observed in whites and Hispanics (Table 5). Since the cause of the steady increase in prevalence every cycle remains unclear and iron status data from NHANES datasets were not utilized, further research including trends in iron deficiency or chronic health conditions [27], widening gap of health disparities [28] or ineffectiveness of current interventions to control anemia could be examined.

The prevalence of anemia increased with ages in men which was consistent with previous findings [13]. In women, there was a bimodal distribution of anemia with peaks in age group 40–49 and over 80 years. The prevalence of anemia for children aged 0.5–4.9 years (3.4%, 95% CI: 2.6–4.3%) was consistent with WHO report for 1999–2005 (3.1%, 95% CI: 2.0–4.7%) and another study for 1999–2002 [25]. However, anemia prevalence in preschool-age black male children was 6.1% (95% CI: 4.1–8.2%), higher than that of both white (2.6%, 95% CI: 0.9–4.2%) and Hispanic (3.7%, 95% CI: 2.3–5.0%) male children in the same age group (Table 4). Similar patterns were observed across races among female children in the pre-school age. These findings were noteworthy when compared to the racial disparities in prevalence of iron deficiency for children between age of 1 and 3 years old [29]. In this age group, twice as many Hispanic children had iron deficiency as black and white children, and Hispanic children were more likely to be overweight and had reduced access to day care, which were identified as risk factors [29]. Compared to age group 0.5–4 years, the prevalence of anemia in age group 5–11 years was significantly higher (3.4 vs 2.0, p = 0.0004), but the difference in prevalence of moderate severe anemia was not statistically significant (p = 0.9884, Table 1). The same patterns of anemia and moderate-severe anemia were observed in these two age groups when separated by gender (Table 3). The analysis by race and gender also showed statistically significant increase in rates of moderate-severe anemia for female blacks from age 0.5–4 years to 5–11 years (0.3 vs 1.7, p = 0.0035, Table 4). Since iron deficiency remains a leading cause of anemia, it was unexpected that the higher prevalence of iron deficiency did not translate to higher rates of anemia. While specific causes of racial/ethnic disparities in anemia are not known, the differences in rates between young children perhaps pointed to a biological explanation or early-development disparities, which require further investigation in future studies.

After the age of 50 years, anemia and moderate-severe anemia prevalence rose rapidly with increasing age (Table 4). For example, 13.6% of black men of age 50–59 years were found to have anemia, and this proportion increased to 41.4% for the 80–85 age group. The prevalence of anemia for individuals between the age of 60 and 85 ranged 6.5%-19.4%, which was higher in comparison to previously measured rates of 10.6% for population over 65 years old in 1988–1994 [13]. Such high rates were significant because anemia is associated with disability and physical decline [30, 31] and anemic older persons over 85 years old have been found to be at risk for higher mortality rates than those without anemia [32]. Moreover, chronic conditions that greatly affect the older population such as cancer and chronic kidney disease can result in anemia and lead to worse prognosis [15, 33, 34]. Thus, anemia in older individuals requires greater public health attention not only for its high prevalence but also for its potential health consequences.

Several elements of the study design might pose limitations in estimating anemia rates. Firstly, small sample sizes did not permit accurate estimates for some age groups of ethnic/racial subgroups. Secondly, low numbers of moderate-severe anemic individuals in subgroups could have affected the estimate as a weighted proportion. Any estimate with small numbers of anemia and/or wide confidence intervals should thus be interpreted carefully. Thirdly, the study did not use any of the iron status data available in the NHANES datasets. Consequently, any speculation about iron deficiency anemia and other causes should be interpreted with caution. Lastly, at the population level, serum Hb concentration in comparison to other clinical measures is the most reliable indicator of anemia. However, anemia is a complex medical condition that results from a multitude of factors. The study estimated rates of anemia based upon WHO definition using Hb levels, which had received calls for adjustments in altitude and smoking behavior [17, 35]. Data in blacks were not race adjusted as recommended by the CDC; therefore, prevalence with adjusted anemia definition would be lower among adult blacks. Moreover, the definition assumed a normal distribution of Hb, which might be negatively skewed as seen in the distribution curve (Fig 2). Overall, these factors might result in over-estimation of prevalence.

## Conclusion

These findings provided an updated snapshot of anemia in the US general population and in subgroups divided by gender, age, race/ethnicity, over years, and severity. The prevalence of anemia has increased over the study period from 2003 to 2012. High risk groups for anemia and moderate-severe anemia include the elderly, reproductive-age and pregnant women, Hispanics, and non-Hispanic blacks.
